# Supplementary material for: MicroRNA regulation of murine trophoblast stem cell self-renewal and differentiation
Source: Life Sci Alliance. 2020 Sep 9;3(11):e202000674. doi: 10.26508/lsa.202000674 (PMC7494815; doi:10.26508/lsa.202000674)
Supplement: Supplementary file 3 [file LSA-2020-00674_SdataF1.pdf]

C.

Source data Figure 1

Replicate 1

	miR-291a-5p	Ct mean	U6 snRNA	Ct mean	ΔCt	ΔΔCt	**RQ ($2^{-\Delta\Delta Ct}$)**
TSC	27.97	27.98	20.79	20.87	7.11	0.00	**1.00**
	27.97		20.85				
	27.99		20.96				
Diff.	30.62	30.64	21.47	21.47	9.17	2.06	**0.24**
	30.56		21.43				
	30.74		21.51				

Replicate 2

	miR-291a-5p	Ct mean	U6 snRNA	Ct mean	ΔCt	ΔΔCt	**RQ ($2^{-\Delta\Delta Ct}$)**
TSC	27.03	27.07	20.87	20.85	6.22	0.00	**1.00**
	27.07		20.89				
	27.12		20.79				
Diff.	29.36	29.28	21.52	21.40	7.88	1.65	**0.32**
	29.25		21.44				
	29.22		21.24				

Replicate 3

	miR-291a-5p	Ct mean	U6 snRNA	Ct mean	ΔCt	ΔΔCt	**RQ ($2^{-\Delta\Delta Ct}$)**
TSC	27.58	27.66	21.26	21.27	6.38	0.00	**1.00**
	27.70		21.34				
	27.69		21.22				
Diff.	30.62	30.64	21.67	21.75	8.89	2.51	**0.18**
	30.56		21.88				
	30.74		21.69				

Replicate 1

	miR-291b-3p	Ct mean	U6 snRNA	Ct mean	ΔCt	ΔΔCt	**RQ ($2^{-\Delta\Delta Ct}$)**
TSC	27.01	27.11	20.70	20.74	6.37	0.00	**1.00**
	27.00		20.78				
	27.32		20.74				
Diff.	28.82	28.95	20.64	20.65	8.30	1.93	**0.26**
	29.04		20.68				
	28.98		20.62				

Replicate 2

	miR-291b-3p	Ct mean	U6 snRNA	Ct mean	ΔCt	ΔΔCt	**RQ ($2^{-\Delta\Delta Ct}$)**
TSC	26.94	26.96	20.93	20.94	6.02	0.00	**1.00**
	26.94		20.92				
	26.99		20.97				
Diff.	29.41	29.38	21.54	21.53	7.85	1.83	**0.28**
	29.35		21.52				
	29.38		21.54				

Replicate 3

	miR-291b-3p	Ct mean	U6 snRNA	Ct mean	ΔCt	ΔΔCt	**RQ ($2^{-\Delta\Delta Ct}$)**
TSC	26.88	26.83	21.46	21.36	5.47	0.00	**1.00**
	26.84		21.34				
	26.78		21.28				
Diff.	29.08	29.12	21.56	21.39	7.73	2.26	**0.21**
	29.12		21.38				
	29.16		21.22				

Replicate 1

	miR-292a-3p	Ct mean	U6 snRNA	Ct mean	ΔCt	ΔΔCt	**RQ ($2^{-\Delta\Delta Ct}$)**
TSC	21.87	21.91	20.96	20.96	0.96	0.00	**1.00**
	21.90		20.96				
	21.97		20.95				
Diff.	24.28	24.32	21.48	21.47	2.84	1.89	**0.27**
	24.31		21.44				
	24.36		21.50				

Replicate 2

	miR-292a-3p	Ct mean	U6 snRNA	Ct mean	ΔCt	ΔΔCt	**RQ ($2^{-\Delta\Delta Ct}$)**
TSC	24.38	24.40	20.94	20.96	3.44	0.00	**1.00**
	24.32		20.98				
	24.50		20.96				
Diff.	25.62	25.67	20.74	20.76	4.91	1.47	**0.36**
	25.76		20.78				
	25.64		20.76				

Replicate 3

	miR-292a-3p	Ct mean	U6 snRNA	Ct mean	ΔCt	ΔΔCt	**RQ ($2^{-\Delta\Delta Ct}$)**
TSC	22.01	22.02	21.32	21.35	0.68	0.00	**1.00**
	22.08		21.34				
	21.98		21.38				
Diff.	24.94	24.94	21.98	21.96	2.98	2.30	**0.20**
	24.97		21.99				
	24.92		21.92				

Replicate 1

	miR-294-3p	Ct mean	U6 snRNA	Ct mean	ΔCt	ΔΔCt	**RQ ($2^{-\Delta\Delta Ct}$)**
TSC	20.91	20.92	20.95	20.88	0.04	0.00	**1.00**
	20.88		20.85				
	20.97		20.85				
Diff.	23.00	23.00	20.06	20.10	2.90	2.86	**0.14**
	22.99		20.07				
	23.02		20.18				

Replicate 2

	miR-294-3p	Ct mean	U6 snRNA	Ct mean	ΔCt	ΔΔCt	**RQ ($2^{-\Delta\Delta Ct}$)**
TSC	20.78	20.79	20.98	20.96	-0.17	0.00	**1.00**
	20.82		20.92				
	20.78		20.98				
Diff.	22.12	22.33	20.16	20.14	2.19	2.36	**0.19**
	22.16		20.12				
	22.72		20.14				

Replicate 3

	miR-294-3p	Ct mean	U6 snRNA	Ct mean	ΔCt	ΔΔCt	**RQ ($2^{-\Delta\Delta Ct}$)**
TSC	21.11	21.15	20.76	20.76	0.39	0.00	**1.00**
	21.08		20.77				
	21.25		20.74				
Diff.	24.34	24.37	20.96	20.93	3.45	3.06	**0.12**
	24.36		20.90				
	24.42		20.92				

Replicate 1

	miR-295-3p	Ct mean	U6 snRNA	Ct mean	ΔCt	ΔΔCt	**RQ ($2^{-\Delta\Delta Ct}$)**
TSC	21.97	21.95	21.44	21.41	0.54	0.00	**1.00**
	21.96		21.38				
	21.93		21.41				
Diff.	25.11	25.14	21.82	21.86	3.28	2.74	**0.15**
	25.07		21.84				
	25.25		21.92				

Replicate 2

	miR-295-3p	Ct mean	U6 snRNA	Ct mean	ΔCt	ΔΔCt	**RQ ($2^{-\Delta\Delta Ct}$)**
TSC	21.46	21.52	20.97	20.89	0.63	0.00	**1.00**
	21.53		20.84				
	21.57		20.86				
Diff.	23.92	23.95	20.99	20.99	2.96	2.33	**0.20**
	23.96		20.95				
	23.98		21.03				

Replicate 3

	miR-295-3p	Ct mean	U6 snRNA	Ct mean	ΔCt	ΔΔCt	**RQ ($2^{-\Delta\Delta Ct}$)**
TSC	21.01	21.02	20.98	21.00	0.03	0.00	**1.00**
	21.08		21.02				
	20.98		20.99				
Diff.	23.34	23.46	21.22	21.25	2.21	2.19	**0.22**
	23.57		21.26				
	23.48		21.27				

D.

Source data Figure 1

Replicate 1

	miR-322-5p	Ct mean	U6 snRNA	Ct mean	ΔCt	ΔΔCt	RQ ($2^{-\Delta\Delta Ct}$)
TSC	22.44	22.47	20.83	20.89	1.58	0.00	**1.00**
	22.43		20.89				
	22.54		20.95				
Diff.	17.97	17.95	20.61	20.66	-2.71	-4.29	**19.52**
	17.91		20.63				
	17.98		20.74				

Replicate 2

	miR-322-5p	Ct mean	U6 snRNA	Ct mean	ΔCt	ΔΔCt	RQ ($2^{-\Delta\Delta Ct}$)
TSC	22.56	22.56	20.87	20.89	1.67	0.00	**1.00**
	22.53		20.94				
	22.58		20.86				
Diff.	18.12	18.17	20.69	20.74	-2.58	-4.24	**18.94**
	18.17		20.65				
	18.21		20.89				

Replicate 3

	miR-322-5p	Ct mean	U6 snRNA	Ct mean	ΔCt	ΔΔCt	RQ ($2^{-\Delta\Delta Ct}$)
TSC	24.32	24.38	20.98	20.94	3.44	0.00	**1.00**
	24.38		20.91				
	24.44		20.94				
Diff.	20.28	20.25	21.14	21.17	-0.92	-4.36	**20.49**
	20.26		21.18				
	20.20		21.18				

Replicate 1

	miR-503-5p	Ct mean	U6 snRNA	Ct mean	ΔCt	ΔΔCt	RQ ($2^{-\Delta\Delta Ct}$)
TSC	29.3	29.38	21.98	21.97	7.41	0.00	**1.00**
	29.42		21.93				
	29.41		21.99				
Diff.	24.92	24.95	20.6	20.69	4.27	-3.14	**8.84**
	24.96		20.71				
	24.98		20.75				

Replicate 2

	miR-503-5p	Ct mean	U6 snRNA	Ct mean	ΔCt	ΔΔCt	RQ ($2^{-\Delta\Delta Ct}$)
TSC	29.78	29.83	21.76	21.95	7.88	0.00	**1.00**
	29.86		21.88				
	29.85		22.20				
Diff.	25.44	25.43	20.95	20.95	4.48	-3.40	**10.56**
	25.44		20.91				
	25.42		20.99				

Replicate 3

	miR-503-5p	Ct mean	U6 snRNA	Ct mean	ΔCt	ΔΔCt	RQ ($2^{-\Delta\Delta Ct}$)
TSC	30.32	30.34	21.67	21.69	8.65	0.00	**1.00**
	30.33		21.75				
	30.37		21.66				
Diff.	27.98	27.95	21.96	21.97	5.97	-2.67	**6.38**
	27.94		21.97				
	27.92		21.99				

Replicate 1

	miR-351-5p	Ct mean	U6 snRNA	Ct mean	ΔCt	ΔΔCt	RQ ($2^{-\Delta\Delta Ct}$)
TSC	27.42	27.45	20.56	20.43	7.02	0.00	**1.00**
	27.44		20.36				
	27.49		20.38				
Diff.	24.92	24.95	20.6	20.69	4.27	-2.75	**6.73**
	24.96		20.71				
	24.98		20.75				

Replicate 2

	miR-351-5p	Ct mean	U6 snRNA	Ct mean	ΔCt	ΔΔCt	RQ ($2^{-\Delta\Delta Ct}$)
TSC	28.22	28.26	21.97	21.96	6.30	0.00	**1.00**
	28.29		21.92				
	28.26		21.98				
Diff.	25.24	25.30	20.85	20.85	4.45	-1.85	**3.61**
	25.44		20.82				
	25.22		20.89				

Replicate 3

	miR-351-5p	Ct mean	U6 snRNA	Ct mean	ΔCt	ΔΔCt	RQ ($2^{-\Delta\Delta Ct}$)
TSC	27.63	27.69	19.94	19.95	7.74	0.00	**1.00**
	27.69		19.94				
	27.76		19.98				
Diff.	25.95	25.96	20.65	20.67	5.29	-2.45	**5.46**
	25.95		20.66				
	25.97		20.69				

Replicate 1

	miR-542-3p	Ct mean	U6 snRNA	Ct mean	ΔCt	ΔΔCt	RQ ($2^{-\Delta\Delta Ct}$)
TSC	32.51	32.59	20.93	20.96	11.63	0.00	**1.00**
	32.53		20.97				
	32.73		20.97				
Diff.	29.03	29.02	21.27	21.28	7.74	-3.89	**14.86**
	28.97		21.29				
	29.06		21.28				

Replicate 2

	miR-542-3p	Ct mean	U6 snRNA	Ct mean	ΔCt	ΔΔCt	RQ ($2^{-\Delta\Delta Ct}$)
TSC	33.43	33.81	21.77	21.74	12.07	0.00	**1.00**
	33.78		21.72				
	34.22		21.74				
Diff.	28.76	28.68	20.65	20.59	8.09	-3.97	**15.71**
	28.65		20.62				
	28.63		20.49				

Replicate 3

	miR-542-3p	Ct mean	U6 snRNA	Ct mean	ΔCt	ΔΔCt	RQ ($2^{-\Delta\Delta Ct}$)
TSC	32.76	32.87	20.55	20.67	12.20	0.00	**1.00**
	32.87		20.73				
	32.98		20.74				
Diff.	29.12	29.27	20.82	20.91	8.36	-3.84	**14.29**
	29.35		20.99				
	29.34		20.92				

Replicate 1

	miR-450b-5p	Ct mean	U6 snRNA	Ct mean	ΔCt	ΔΔCt	RQ ($2^{-\Delta\Delta Ct}$)
TSC	35.95	35.51	17.99	18.00	17.51	0.00	**1.00**
	35.16		17.92				
	35.41		18.08				
Diff.	31.57	31.57	18.29	18.29	13.28	-4.23	**18.77**
	31.59		18.23				
	31.55		18.35				

Replicate 2

	miR-450b-5p	Ct mean	U6 snRNA	Ct mean	ΔCt	ΔΔCt	RQ ($2^{-\Delta\Delta Ct}$)
TSC	37.07	36.82	20.34	20.42	16.40	0.00	**1.00**
	36.86		20.44				
	36.53		20.47				
Diff.	31.44	31.50	19.59	19.58	11.92	-4.49	**22.42**
	31.64		19.54				
	31.42		19.62				

Replicate 3

	miR-450b-5p	Ct mean	U6 snRNA	Ct mean	ΔCt	ΔΔCt	RQ ($2^{-\Delta\Delta Ct}$)
TSC	31.56	31.99	20.45	20.55	11.44	0.00	**1.00**
	32.17		20.54				
	32.23		20.66				
Diff.	28.87	28.23	20.78	20.77	7.46	-3.98	**15.78**
	27.83		20.82				
	27.99		20.72				
