# Supplementary material for: MicroRNA regulation of murine trophoblast stem cell self-renewal and differentiation
Source: Life Sci Alliance. 2020 Sep 9;3(11):e202000674. doi: 10.26508/lsa.202000674 (PMC7494815; doi:10.26508/lsa.202000674)

A.

Source data Figure 2

Replicate 1

	p21	Ct mean	Rpl7	Ct mean	ΔCt	ΔΔCt	RQ ($2^{-\Delta\Delta Ct}$)
Scrambled	22.27	22.31	14.4	14.49	7.82	0.00	**1.00**
	22.43		14.44				
	22.24		14.64				
miR-291b mimic	22.55	22.49	14.43	14.49	8.00	0.18	**0.88**
	22.55		14.46				
	22.38		14.58				
miR-291b inhibitor	21.53	21.43	14.2	14.35	7.08	-0.74	**1.67**
	21.41		14.49				
	21.34		14.35				
miR-292a mimic	22.93	22.60	14.42	14.35	8.25	0.43	**0.74**
	22.76		14.31				
	22.12		14.32				
miR-292a inhibitor	22.08	22.07	14.94	14.68	7.39	-0.43	**1.34**
	22.03		14.76				
	22.1		14.33				
miR-294 mimic	22.96	23.00	14.37	14.49	8.51	0.69	**0.62**
	23		14.59				
	23.03		14.51				
miR-294 inhibitor	21.22	21.35	14.31	14.32	7.02	-0.80	**1.74**
	21.37		14.38				
	21.45		14.28				
miR-295 mimic	23.5	23.30	14.05	14.00	9.31	1.49	**0.36**
	23.45		13.87				
	22.96		14.07				
miR-295 inhibitor	21.55	21.56	14.4	14.69	6.87	-0.95	**1.93**
	21.62		14.71				
	21.5		14.95				

Replicate 2

	p21	Ct mean	Rpl7	Ct mean	ΔCt	ΔΔCt	RQ ($2^{-\Delta\Delta Ct}$)
Scrambled	25.49	25.50	15.28	15.29	10.21	0.00	**1.00**
	25.44		15.37				
	25.57		15.22				
miR-291b mimic	25.93	26.11	15.41	15.28	10.83	0.62	**0.65**
	26.13		15.22				
	26.27		15.2				
miR-291b inhibitor	24.93	24.90	15.21	15.29	9.61	-0.60	**1.51**
	24.78		15.45				
	25		15.21				
miR-292a mimic	25.86	25.82	14.98	14.93	10.89	0.68	**0.63**
	25.76		14.92				
	25.83		14.89				
miR-292a inhibitor	24.98	25.11	15.32	15.39	9.72	-0.49	**1.40**
	25.65		15.43				
	24.7		15.42				
miR-294 mimic	26.21	26.10	15.65	15.63	10.47	0.26	**0.84**
	25.93		15.62				
	26.15		15.61				
miR-294 inhibitor	24.83	24.46	15.44	15.50	8.96	-1.25	**2.37**
	24.92		15.47				
	23.64		15.59				
miR-295 mimic	25.99	25.94	15.29	15.30	10.64	0.43	**0.74**
	25.72		15.34				
	26.1		15.27				
miR-295 inhibitor	24.91	25.32	16.37	16.40	8.92	-1.29	**2.45**
	25.46		16.36				
	25.58		16.46				

Replicate 3

	p21	Ct mean	Rpl7	Ct mean	ΔCt	ΔΔCt	RQ ($2^{-\Delta\Delta Ct}$)
Scrambled	25.1	25.07	15.5	15.56	9.51	0.00	**1.00**
	25.04		15.6				
	25.07		15.57				
miR-291b mimic	25.12	25.50	15.78	15.71	9.78	0.27	**0.83**
	25.78		15.67				
	25.59		15.69				
miR-291b inhibitor	24.2	24.41	15.93	15.79	8.61	-0.90	**1.87**
	24.65		15.67				
	24.37		15.78				
miR-292a mimic	25.47	25.40	15.77	15.70	9.69	0.18	**0.88**
	25.35		15.71				
	25.37		15.63				
miR-292a inhibitor	24.83	24.48	15.92	15.93	8.55	-0.96	**1.95**
	23.82		15.99				
	24.79		15.87				
miR-294 mimic	26	26.11	15.68	15.61	10.50	0.99	**0.50**
	25.99		15.53				
	26.33		15.61				
miR-294 inhibitor	24.17	24.23	15.44	15.60	8.63	-0.89	**1.85**
	24.16		15.79				
	24.35		15.57				
miR-295 mimic	25.88	26.04	15.84	15.84	10.20	0.68	**0.62**
	26.05		15.87				
	26.19		15.82				
miR-295 inhibitor	24.09	24.06	15.78	15.89	8.17	-1.35	**2.54**
	24.22		15.98				
	23.87		15.92				

Replicate 1

	p27	Ct mean	Rpl7	Ct mean	ΔCt	ΔΔCt	RQ ($2^{-\Delta\Delta Ct}$)
Scrambled	25.85	25.85	12.35	12.34	13.51	0.00	**1.00**
	25.78		12.27				
	25.93		12.41				
miR-294 mimic	26.17	26.24	12.68	12.47	13.78	0.27	**0.83**
	26.25		12.41				
	26.31		12.31				
miR-294 inhibitor	25.53	25.59	13.11	12.85	12.74	-0.77	**1.70**
	25.92		12.71				
	25.32		12.72				
miR-295 mimic	26.26	26.37	12.7	12.71	13.66	0.15	**0.90**
	26.36		12.62				
	26.49		12.82				
miR-295 inhibitor	24.76	25.03	12.23	12.27	12.77	-0.74	**1.67**
	24.95		12.03				
	25.39		12.54				

Replicate 2

	p27	Ct mean	Rpl7	Ct mean	ΔCt	ΔΔCt	RQ ($2^{-\Delta\Delta Ct}$)
Scrambled	27.42	27.38	15.38	15.41	11.97	0.00	**1.00**
	27.34		15.42				
	27.38		15.44				
miR-294 mimic	27.42	28.03	15.43	15.41	12.62	0.65	**0.64**
	28.33		15.47				
	28.33		15.32				
miR-294 inhibitor	26.52	26.59	15.3	15.24	11.35	-0.62	**1.53**
	26.59		15.21				
	26.67		15.22				
miR-295 mimic	28.12	28.30	15.58	15.55	12.75	0.78	**0.58**
	28.53		15.61				
	28.24		15.46				
miR-295 inhibitor	26.99	27.16	15.77	15.70	11.46	-0.51	**1.42**
	27.36		15.53				
	27.12		15.79				

Replicate 3

	p27	Ct mean	Rpl7	Ct mean	ΔCt	ΔΔCt	RQ ($2^{-\Delta\Delta Ct}$)
Scrambled	27.1	26.97	14.98	14.98	11.99	0.00	**1.00**
	26.88		14.91				
	26.92		15.04				
miR-294 mimic	27.96	27.89	15.32	15.19	12.71	0.72	**0.61**
	27.83		15.01				
	27.89		15.23				
miR-294 inhibitor	26.64	26.75	15.37	15.30	11.45	-0.54	**1.46**
	26.88		15.39				
	26.73		15.15				
miR-295 mimic	28.03	28.40	15.72	15.72	12.68	0.69	**0.62**
	28.34		15.76				
	28.82		15.68				
miR-295 inhibitor	27.83	27.24	15.78	15.75	11.49	-0.50	**1.42**
	26.92		15.73				
	26.97		15.75				

A. (Cont...)

Source data Figure 2

Replicate 1

	Wee1	Ct mean	Rpl7	Ct mean	ΔCt	ΔΔCt	RQ ($2^{-\Delta\Delta Ct}$)
Scrambled	22.34	22.35	16.35	16.34	6.01	0.00	**1.00**
	22.29		16.26				
	22.42		16.42				
miR-291b mimic	22.59	22.69	15.76	15.80	6.89	0.89	**0.54**
	22.69		15.72				
	22.8		15.92				
miR-291b inhibitor	20.5	20.47	16.28	16.40	4.07	-1.93	**3.82**
	20.5		16.39				
	20.41		16.52				
miR-294 mimic	23.25	23.34	16.96	17.29	6.05	0.04	**0.97**
	23.29		16.98				
	23.47		17.93				
miR-294 inhibitor	21	21.00	16.46	16.37	4.63	-1.38	**2.60**
	21.05		16.27				
	20.96		16.39				
miR-295 mimic	22.52	22.64	16.25	16.38	6.26	0.25	**0.84**
	22.51		16.41				
	22.88		16.47				
miR-295 inhibitor	20.89	20.88	16.2	16.32	4.56	-1.45	**2.73**
	20.83		16.27				
	20.92		16.5				

Replicate 2

	Wee1	Ct mean	Rpl7	Ct mean	ΔCt	ΔΔCt	RQ ($2^{-\Delta\Delta Ct}$)
Scrambled	21.89	22.06	15.88	15.71	6.34	0.00	**1.00**
	22.21		15.72				
	22.07		15.54				
miR-291b mimic	22.75	22.19	15.43	15.56	6.63	0.29	**0.82**
	22.59		15.69				
	21.24		15.56				
miR-291b inhibitor	20.18	20.39	15.87	15.84	4.56	-1.79	**3.45**
	20.77		15.83				
	20.23		15.81				
miR-294 mimic	22.21	22.27	15.22	15.35	6.93	0.58	**0.67**
	22.3		15.31				
	22.31		15.51				
miR-294 inhibitor	20.72	20.78	15.77	15.70	5.09	-1.26	**2.39**
	20.22		15.63				
	21.41		15.69				
miR-295 mimic	21.94	21.91	15.48	15.49	6.42	0.08	**0.95**
	21.93		15.52				
	21.85		15.46				
miR-295 inhibitor	20.43	20.63	15.86	15.82	4.81	-1.53	**2.89**
	20.63		15.79				
	20.84		15.82				

Replicate 3

	Wee1	Ct mean	Rpl7	Ct mean	ΔCt	ΔΔCt	RQ ($2^{-\Delta\Delta Ct}$)
Scrambled	27.1	26.97	15.21	15.27	11.70	0.00	**1.00**
	26.88		15.35				
	26.92		15.24				
miR-291b mimic	27	27.08	15.21	15.27	11.81	0.11	**0.92**
	27.04		15.28				
	27.21		15.32				
miR-291b inhibitor	25.13	25.22	15.67	15.63	9.59	-2.11	**4.32**
	25.22		15.62				
	25.3		15.59				
miR-294 mimic	27.23	27.61	15.73	15.74	11.87	0.17	**0.89**
	27.92		15.71				
	27.68		15.78				
miR-294 inhibitor	25.27	25.41	15.55	15.40	10.01	-1.69	**3.22**
	25.76		15.38				
	25.21		15.27				
miR-295 mimic	26.9	27.22	15.31	15.38	11.84	0.14	**0.91**
	26.92		15.47				
	27.83		15.36				
miR-295 inhibitor	25.76	25.64	15.22	15.23	10.41	-1.29	**2.44**
	25.34		15.19				
	25.83		15.28				

Replicate 1

	Rbl2	Ct mean	Rpl7	Ct mean	ΔCt	ΔΔCt	RQ ($2^{-\Delta\Delta Ct}$)
Scrambled	27.44	27.61	15.97	15.95	11.66	0.00	**1.00**
	27.47		15.94				
	27.92		15.95				
miR-291b mimic	28.76	28.84	15.77	15.90	12.93	1.28	**0.41**
	28.82		16.03				
	28.93		15.91				
miR-291b inhibitor	25.87	25.87	15.84	15.90	9.96	-1.69	**3.23**
	25.91		15.9				
	25.82		15.97				

Replicate 2

	Rbl2	Ct mean	Rpl7	Ct mean	ΔCt	ΔΔCt	RQ ($2^{-\Delta\Delta Ct}$)
Scrambled	25.49	25.50	16.23	16.14	9.36	0.00	**1.00**
	25.44		16.17				
	25.57		16.03				
miR-291b mimic	26.22	26.22	15.98	16.03	10.19	0.83	**0.56**
	26.15		16.02				
	26.29		16.1				
miR-291b inhibitor	23.2	23.68	16.31	16.24	7.44	-1.92	**3.78**
	23.93		16.25				
	23.91		16.16				

Replicate 3

	Rbl2	Ct mean	Rpl7	Ct mean	ΔCt	ΔΔCt	RQ ($2^{-\Delta\Delta Ct}$)
Scrambled	25.51	25.50	15.76	15.63	9.87	0.00	**1.00**
	25.42		15.36				
	25.57		15.77				
miR-291b mimic	27.46	27.55	15.79	15.70	11.86	1.99	**0.25**
	27.45		15.69				
	27.75		15.61				
miR-291b inhibitor	23.68	23.72	15.54	15.56	8.16	-1.71	**3.26**
	23.75		15.55				
	23.74		15.59				

Replicate 1

	E2f7	Ct mean	Rpl7	Ct mean	ΔCt	ΔΔCt	RQ ($2^{-\Delta\Delta Ct}$)
Scrambled	25.39	25.37	15.76	15.63	9.74	0.00	**1.00**
	25.45		15.36				
	25.27		15.77				
miR-291b mimic	25.68	25.67	15.7	15.66	10.01	0.27	**0.83**
	25.63		15.58				
	25.69		15.7				
miR-291b inhibitor	23.12	23.06	15.59	15.56	7.50	-2.24	**4.71**
	23.16		15.5				
	22.91		15.59				
miR-294 mimic	25.72	25.80	15.49	15.48	10.33	0.59	**0.67**
	25.76		15.45				
	25.93		15.49				
miR-294 inhibitor	22.87	23.11	15.52	15.66	7.45	-2.29	**4.89**
	23.53		15.94				
	22.93		15.52				
miR-295 mimic	25.89	25.98	15.59	15.55	10.43	0.69	**0.62**
	25.93		15.46				
	26.13		15.61				
miR-295 inhibitor	23.17	23.23	15.44	15.53	7.70	-2.04	**4.10**
	23.13		15.69				
	23.39		15.45				

Replicate 2

	E2f7	Ct mean	Rpl7	Ct mean	ΔCt	ΔΔCt	RQ ($2^{-\Delta\Delta Ct}$)
Scrambled	26.26	26.28	15.11	15.15	11.13	0.00	**1.00**
	26.21		15.09				
	26.37		15.24				
miR-291b mimic	26.53	26.77	15.58	15.55	11.23	0.09	**0.94**
	27.03		15.57				
	26.76		15.49				
miR-291b inhibitor	24.98	25.09	15.82	15.85	9.24	-1.89	**3.71**
	25.21		15.91				
	25.07		15.81				
miR-294 mimic	26.73	26.81	14.97	15.07	11.74	0.61	**0.66**
	26.81		15.02				
	26.89		15.21				
miR-294 inhibitor	24	24.66	15.69	15.72	8.93	-2.20	**4.59**
	25.21		15.73				
	24.76		15.75				
miR-295 mimic	27.59	27.77	15.5	15.47	12.30	1.16	**0.45**
	27.95		15.48				
	27.77		15.44				
miR-295 inhibitor	24.63	24.44	15.76	15.72	8.72	-2.42	**5.34**
	24.57		15.54				
	24.11		15.86				

Replicate 3

	E2f7	Ct mean	Rpl7	Ct mean	ΔCt	ΔΔCt	RQ ($2^{-\Delta\Delta Ct}$)
Scrambled	27.1	26.97	14.95	14.90	12.07	0.00	**1.00**
	26.88		14.83				
	26.92		14.91				
miR-291b mimic	28.15	27.97	15.41	15.52	12.44	0.37	**0.77**
	27.94		15.52				
	27.81		15.64				
miR-291b inhibitor	24.73	24.85	14.96	14.96	9.89	-2.18	**4.53**
	24.52		15.01				
	25.31		14.92				
miR-294 mimic	27.94	28.17	15.92	15.99	12.17	0.10	**0.93**
	28.64		16.18				
	27.92		15.88				
miR-294 inhibitor	24.69	24.92	15.21	15.28	9.64	-2.43	**5.38**
	24.73		15.37				
	25.35		15.26				
miR-295 mimic	29.76	29.31	15.63	15.31	14.00	1.93	**0.26**
	29.49		14.92				
	28.68		15.37				
miR-295 inhibitor	25.87	25.39	15.58	15.49	9.90	-2.17	**4.50**
	24.93		15.38				
	25.38		15.52				

B.

Source data Figure 2

For Figure 2B, P21 and RPL7

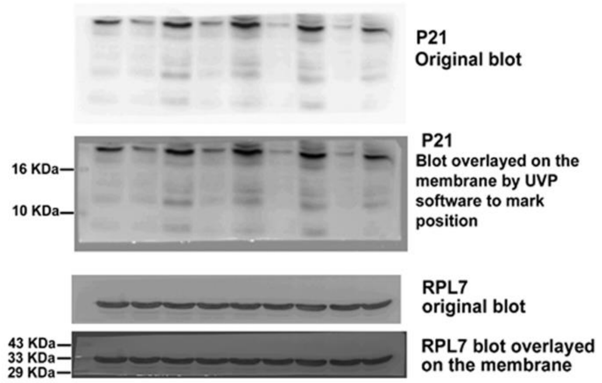

For Figure 2B, RBL2 and RPL7

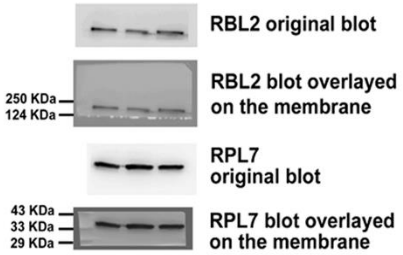

For Figure 2B, P27 and RPL7

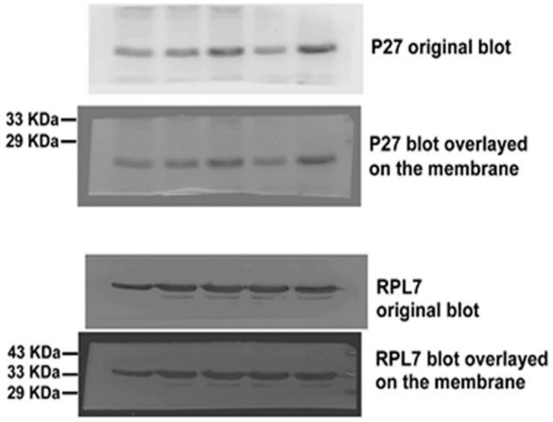

For Figure 2B, E2F7 and RPL7

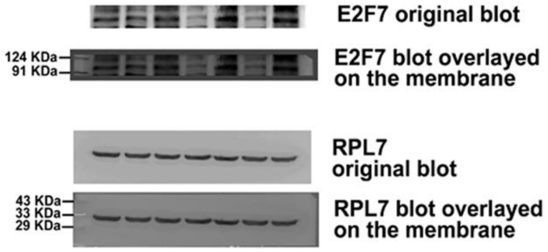

For Figure 2B, WEE1 and RPL7

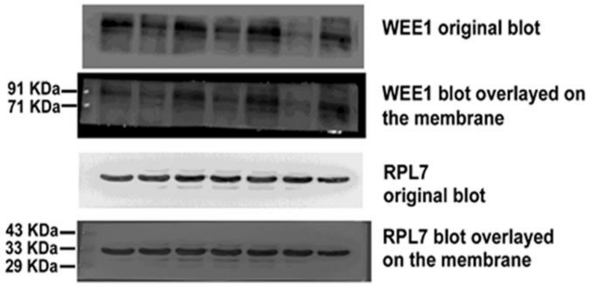

Source data Figure 2

C.

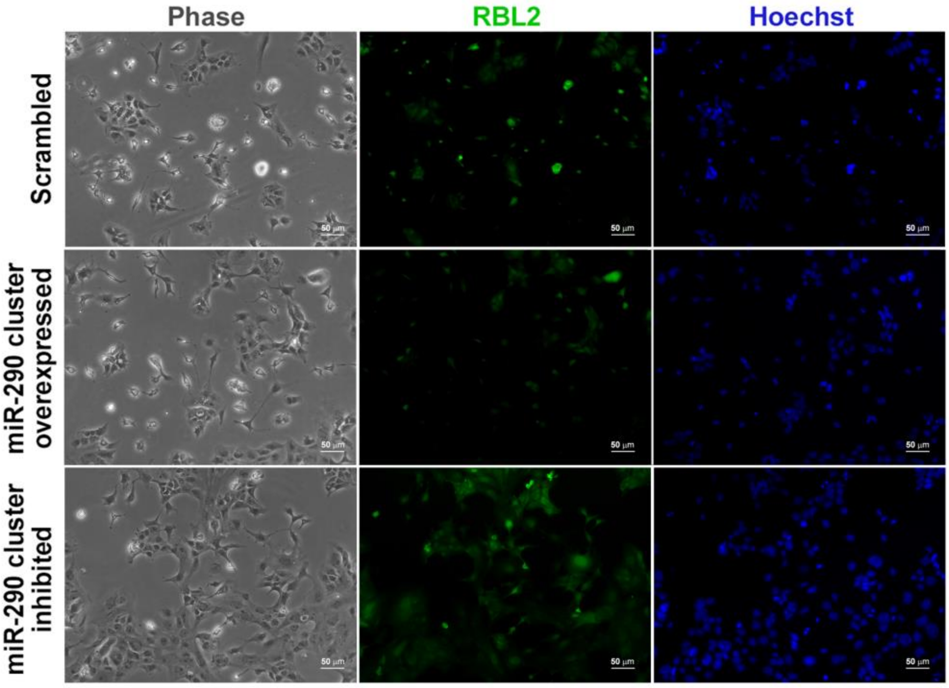

Supplement: Supplementary file 5 [file LSA-2020-00674_SdataF2.pdf]
