# Supplementary material for: MicroRNA regulation of murine trophoblast stem cell self-renewal and differentiation
Source: Life Sci Alliance. 2020 Sep 9;3(11):e202000674. doi: 10.26508/lsa.202000674 (PMC7494815; doi:10.26508/lsa.202000674)

A.

Source data Figure 3

Replicate 1

	Cyclin D1	Ct mean	Rpl7	Ct mean	ΔCt	ΔΔCt	RQ ($2^{-\Delta\Delta Ct}$)
Scrambled	19.87	20.07	13.39	13.15	6.92	0.00	**1.00**
	20.1		12.99				
	20.24		13.08				
miR-322 mimic	23.11	23.24	13.14	13.31	9.93	3.01	**0.12**
	23.72		13.03				
	22.9		13.77				
miR-322 inhibitor	19.8	19.98	13.23	13.11	6.87	-0.04	**1.03**
	20.21		13.15				
	19.94		12.95				
miR-503 mimic	22.84	22.68	13.03	13.32	9.35	2.44	**0.18**
	22.67		13.37				
	22.52		13.57				
miR-503 inhibitor	19.88	19.88	13.35	13.46	6.42	-0.49	**1.41**
	19.87		13.58				
	19.89		13.44				

Replicate 2

	Cyclin D1	Ct mean	Rpl7	Ct mean	ΔCt	ΔΔCt	RQ ($2^{-\Delta\Delta Ct}$)
Scrambled	20.68	20.54	15.88	15.78	4.76	0.00	**1.00**
	20.52		15.78				
	20.42		15.67				
miR-322 mimic	22.12	22.42	15.17	15.47	6.95	2.19	**0.22**
	22.79		15.62				
	22.35		15.61				
miR-322 inhibitor	19.63	19.75	15.2	14.94	4.81	0.05	**0.97**
	19.75		14.73				
	19.88		14.89				
miR-503 mimic	22.92	22.76	15.72	15.77	6.98	2.22	**0.21**
	22.76		15.77				
	22.59		15.83				
miR-503 inhibitor	20.06	19.89	15.35	15.48	4.42	-0.35	**1.27**
	20.14		15.75				
	19.48		15.33				

Replicate 3

	Cyclin D1	Ct mean	Rpl7	Ct mean	ΔCt	ΔΔCt	RQ ($2^{-\Delta\Delta Ct}$)
Scrambled	21.35	21.41	15.89	15.78	5.63	0.00	**1.00**
	21.42		15.72				
	21.46		15.72				
miR-322 mimic	22.28	22.09	15.37	15.18	6.91	1.28	**0.41**
	22.07		14.58				
	21.92		15.59				
miR-322 inhibitor	20.94	20.25	14.98	14.97	5.28	-0.35	**1.28**
	20.58		14.9				
	19.23		15.03				
miR-503 mimic	23.01	23.14	15.79	15.29	7.85	2.21	**0.22**
	23.19		15.02				
	23.22		15.07				
miR-503 inhibitor	20.23	20.23	15.04	14.83	5.40	-0.23	**1.18**
	20.18		15.18				
	20.28		14.27				

Replicate 1

	Cyclin E1	Ct mean	Rpl7	Ct mean	ΔCt	ΔΔCt	RQ ($2^{-\Delta\Delta Ct}$)
Scrambled	20.1	20.21	14.11	14.74	5.47	0.00	**1.00**
	20.29		14.28				
	20.25		15.84				
miR-322 mimic	20.47	20.42	14.05	14.41	6.02	0.55	**0.68**
	20.31		14.89				
	20.49		14.28				
miR-322 inhibitor	19.75	19.82	14.51	14.64	5.18	-0.29	**1.22**
	19.83		14.49				
	19.87		14.91				
miR-503 mimic	20.31	20.23	14.58	14.51	5.73	0.26	**0.84**
	20.23		14.5				
	20.16		14.44				
miR-503 inhibitor	20.35	19.85	14.55	14.62	5.23	-0.24	**1.18**
	19.34		14.86				
	19.87		14.46				

Replicate 2

	Cyclin E1	Ct mean	Rpl7	Ct mean	ΔCt	ΔΔCt	RQ ($2^{-\Delta\Delta Ct}$)
Scrambled	20.76	20.77	15.55	15.69	5.08	0.00	**1.00**
	20.81		15.73				
	20.74		15.78				
miR-322 mimic	21.12	21.21	15.82	15.81	5.40	0.32	**0.80**
	21.45		15.73				
	21.07		15.89				
miR-322 inhibitor	19.82	20.13	15.67	15.58	4.55	-0.53	**1.45**
	20.45		15.49				
	20.12		15..63				
miR-503 mimic	20.98	21.06	15.84	15.87	5.18	0.10	**0.93**
	20.86		15.96				
	21.33		15.82				
miR-503 inhibitor	20.17	20.34	15.95	15.80	4.54	-0.54	**1.46**
	20.01		15.77				
	20.84		15.68				

Replicate 3

	Cyclin E1	Ct mean	Rpl7	Ct mean	ΔCt	ΔΔCt	RQ ($2^{-\Delta\Delta Ct}$)
Scrambled	21.22	21.27	15.07	15.24	6.03	0.00	**1.00**
	21.2		15.36				
	21.38		15.29				
miR-322 mimic	22.78	22.34	15.43	15.62	6.72	0.70	**0.62**
	22.17		15.73				
	22.07		15.69				
miR-322 inhibitor	20.76	20.86	15.35	15.44	5.42	-0.60	**1.52**
	20.88		15.57				
	20.94		15.39				
miR-503 mimic	21.32	21.31	15.26	15.34	5.97	-0.06	**1.04**
	21.37		15.26				
	21.23		15.49				
miR-503 inhibitor	20.97	20.67	14.81	14.97	5.69	-0.33	**1.26**
	20.38		15.08				
	20.65		15.03				

Replicate 1

	Cdc25b	Ct mean	Rpl7	Ct mean	ΔCt	ΔΔCt	RQ ($2^{-\Delta\Delta Ct}$)
Scrambled	25.81	25.88	14.87	14.91	10.97	0.00	**1.00**
	25.92		14.92				
	25.92		14.95				
miR-322 mimic	29.49	29.58	14.95	14.85	14.73	3.76	**0.07**
	29.54		14.77				
	29.7		14.82				
miR-322 inhibitor	24.78	24.86	14.74	14.77	10.09	-0.88	**1.84**
	24.88		14.79				
	24.93		14.78				

Replicate 2

	Cdc25b	Ct mean	Rpl7	Ct mean	ΔCt	ΔΔCt	RQ ($2^{-\Delta\Delta Ct}$)
Scrambled	26.82	26.67	15.23	15.31	11.36	0.00	**1.00**
	26.39		15.44				
	26.79		15.26				
miR-322 mimic	29.12	28.91	15.18	15.06	13.85	2.49	**0.18**
	28.88		14.93				
	28.72		15.07				
miR-322 inhibitor	25.98	25.96	15.18	15.20	10.76	-0.60	**1.52**
	26		15.21				
	25.9		15.22				

Replicate 3

	Cdc25b	Ct mean	Rpl7	Ct mean	ΔCt	ΔΔCt	RQ ($2^{-\Delta\Delta Ct}$)
Scrambled	25.76	25.80	15.77	15.71	10.09	0.00	**1.00**
	25.94		15.68				
	25.7		15.69				
miR-322 mimic	29.03	29.14	15.33	15.32	13.82	3.74	**0.08**
	29.23		15.28				
	29.17		15.35				
miR-322 inhibitor	25.01	24.92	15.73	15.67	9.25	-0.83	**1.78**
	24.84		15.56				
	24.92		15.72				

Source data Figure 3

B.

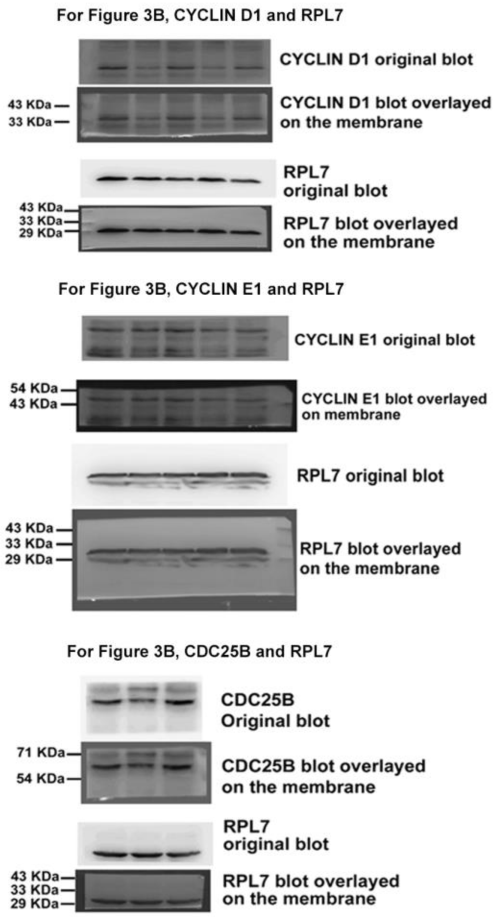

C.

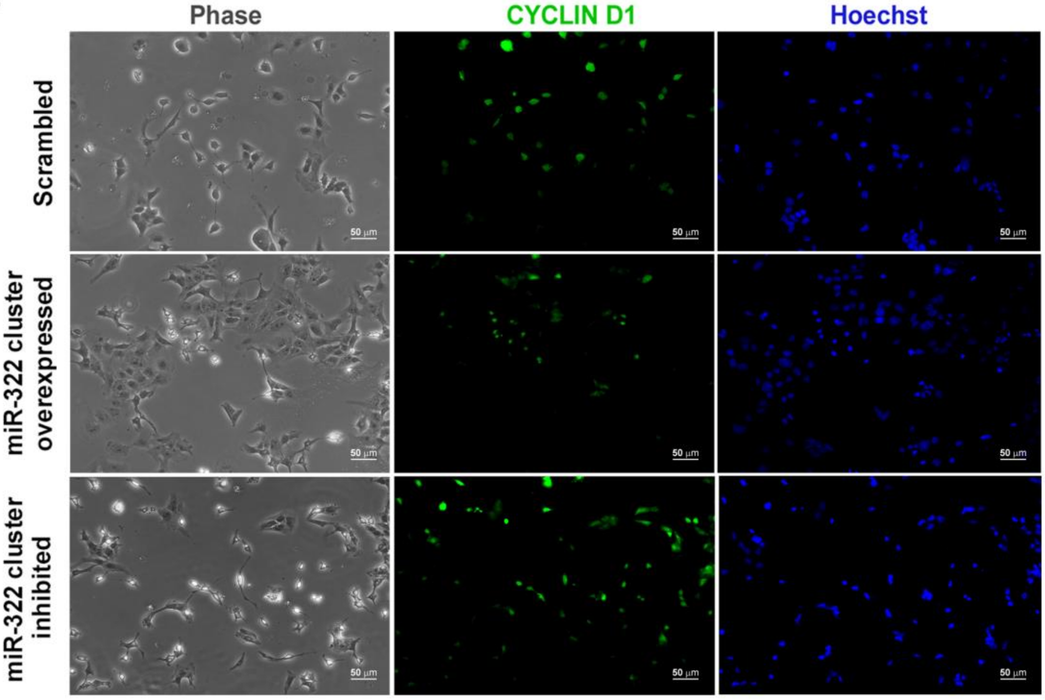

Supplement: Supplementary file 6 [file LSA-2020-00674_SdataF3.pdf]
