# Supplementary material for: MicroRNA regulation of murine trophoblast stem cell self-renewal and differentiation
Source: Life Sci Alliance. 2020 Sep 9;3(11):e202000674. doi: 10.26508/lsa.202000674 (PMC7494815; doi:10.26508/lsa.202000674)

Source data Figure 4

A.

BrdU Incorporation Assay

5000 cells

	**Replicate 1**		**Replicate 2**		**Replicate 3**		**Replicate 4**	
	Mean OD	Rel. incorporation	Mean OD	Rel. incorporation	Mean OD	Rel. incorporation	Mean OD	Rel. incorporation
Scrambled	1.956	1.000	1.693	1.000	1.743	1.000	1.874	1.000
miR-290 cluster inhibited	0.403	0.206	1.045	0.617	1.036	0.594	1.054	0.562
miR-322 cluster overexpressed	1.118	0.572	0.993	0.587	0.367	0.211	1.023	0.546

10000 cells

	**Replicate 1**		**Replicate 2**		**Replicate 3**		**Replicate 4**	
	Mean OD	Rel. incorporation	Mean OD	Rel. incorporation	Mean OD	Rel. incorporation	Mean OD	Rel. incorporation
Scrambled	1.981	1.000	2.24	1.000	2.432	1.000	2.299	1.000
miR-290 cluster inhibited	1.1	0.555	1.086	0.485	0.374	0.154	1.265	0.550
miR-322 cluster overexpressed	0.703	0.355	0.854	0.381	0.783	0.322	0.745	0.324

B.

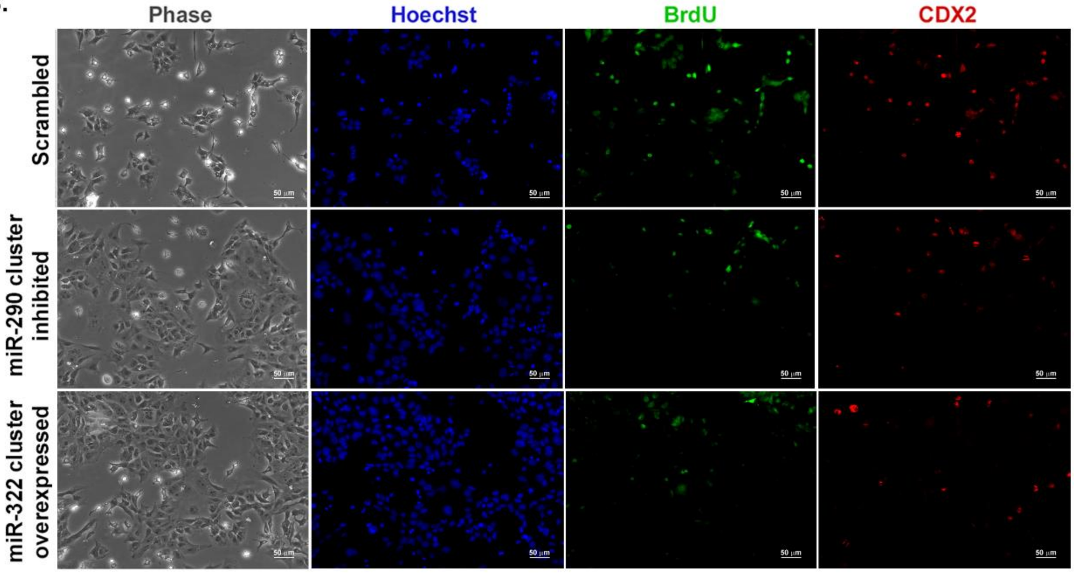

D.

Source data Figure 4

Replicate 1

	Cdx2	Ct mean	Rpl7	Ct mean	ΔCt	ΔΔCt	RQ ($2^{-\Delta\Delta Ct}$)
Scrambled	21.93	21.90	15.9	15.89	6.01	0.00	**1.00**
	21.83		15.81				
	21.94		15.96				
290-cluster inhibited	22.9	23.10	15.76	15.79	7.31	1.30	**0.41**
	23.06		15.74				
	23.34		15.86				

Replicate 2

	Cdx2	Ct mean	Rpl7	Ct mean	ΔCt	ΔΔCt	RQ ($2^{-\Delta\Delta Ct}$)
Scrambled	21.43	21.49	15.26	15.19	6.30	0.00	**1.00**
	21.48		15.18				
	21.56		15.12				
290-cluster inhibited	23.86	23.86	15.99	15.98	7.88	1.57	**0.34**
	23.82		15.98				
	23.90		15.98				

Replicate 3

	Cdx2	Ct mean	Rpl7	Ct mean	ΔCt	ΔΔCt	RQ ($2^{-\Delta\Delta Ct}$)
Scrambled	22.21	22.19	15.23	15.21	6.99	0.00	**1.00**
	22		15.2				
	22.37		15.19				
290-cluster inhibited	24.02	24.20	14.75	14.98	9.22	2.24	**0.21**
	24.26		15.06				
	24.32		15.12				

Replicate 1

	Eomes	Ct mean	Rpl7	Ct mean	ΔCt	ΔΔCt	RQ ($2^{-\Delta\Delta Ct}$)
Scrambled	19.97	20.02	15.92	15.85	4.17	0.00	**1.00**
	19.74		15.83				
	20.36		15.8				
290-cluster inhibited	21.1	21.65	15.85	15.90	5.75	1.58	**0.33**
	21.92		15.97				
	21.93		15.87				

Replicate 2

	Eomes	Ct mean	Rpl7	Ct mean	ΔCt	ΔΔCt	RQ ($2^{-\Delta\Delta Ct}$)
Scrambled	20.12	20.18	14.84	15.09	5.09	0.00	**1.00**
	20.18		14.98				
	20.24		15.44				
290-cluster inhibited	22.78	22.70	15.78	15.76	6.94	1.85	**0.28**
	22.69		15.72				
	22.63		15.78				

Replicate 3

	Eomes	Ct mean	Rpl7	Ct mean	ΔCt	ΔΔCt	RQ ($2^{-\Delta\Delta Ct}$)
Scrambled	21.34	21.29	15.28	15.25	6.04	0.00	**1.00**
	21.28		15.26				
	21.26		15.22				
290-cluster inhibited	23.97	23.99	15.73	15.73	8.26	2.22	**0.21**
	24.01		15.77				
	23.99		15.69				

Replicate 1

	Esrrb	Ct mean	Rpl7	Ct mean	ΔCt	ΔΔCt	RQ ($2^{-\Delta\Delta Ct}$)
Scrambled	21.82	21.74	14.88	14.88	6.86	0.00	**1.00**
	21.54		14.85				
	21.87		14.92				
290-cluster inhibited	23.97	24.12	15.55	15.63	8.49	1.63	**0.32**
	24.06		15.74				
	24.32		15.59				

Replicate 2

	Esrrb	Ct mean	Rpl7	Ct mean	ΔCt	ΔΔCt	RQ ($2^{-\Delta\Delta Ct}$)
Scrambled	22.75	22.46	15.62	15.68	6.78	0.00	**1.00**
	22.78		15.78				
	21.86		15.64				
290-cluster inhibited	23.72	23.67	15.94	15.97	7.70	0.91	**0.53**
	23.68		15.99				
	23.6		15.98				

Replicate 3

	Esrrb	Ct mean	Rpl7	Ct mean	ΔCt	ΔΔCt	RQ ($2^{-\Delta\Delta Ct}$)
Scrambled	21.36	21.43	15.88	15.82	5.60	0.00	**1.00**
	21.45		15.83				
	21.47		15.76				
290-cluster inhibited	23.91	23.63	15.65	15.64	7.99	2.39	**0.19**
	23.98		15.69				
	23.01		15.58				

Replicate 1

	Plf	Ct mean	Rpl7	Ct mean	ΔCt	ΔΔCt	RQ ($2^{-\Delta\Delta Ct}$)
Scrambled	23.69	23.69	15.57	15.57	8.12	0.00	**1.00**
	23.6		15.56				
	23.79		15.58				
290-cluster inhibited	22.92	22.91	15.8	15.84	7.07	-1.05	**2.07**
	22.87		15.78				
	22.95		15.94				

Replicate 2

	Plf	Ct mean	Rpl7	Ct mean	ΔCt	ΔΔCt	RQ ($2^{-\Delta\Delta Ct}$)
Scrambled	23.12	23.11	15.77	15.78	7.33	0.00	**1.00**
	23.17		15.81				
	23.04		15.76				
290-cluster inhibited	22.56	22.52	16.14	16.20	6.32	-1.01	**2.01**
	22.69		16.22				
	22.32		16.25				

Replicate 3

	Plf	Ct mean	Rpl7	Ct mean	ΔCt	ΔΔCt	RQ ($2^{-\Delta\Delta Ct}$)
Scrambled	24.76	24.74	15.23	15.21	9.54	0.00	**1.00**
	24.79		15.2				
	24.68		15.19				
290-cluster inhibited	23.91	23.97	15.99	16.06	7.91	-1.63	**3.09**
	23.96		16.18				
	24.04		16.01				

Replicate 1

	Pl1	Ct mean	Rpl7	Ct mean	ΔCt	ΔΔCt	RQ ($2^{-\Delta\Delta Ct}$)
Scrambled	25.1	25.10	14.94	14.93	10.16	0.00	**1.00**
	24.97		14.94				
	25.22		14.92				
290-cluster inhibited	21.91	21.97	14.73	14.67	7.30	-2.86	**7.26**
	22.06		14.53				
	21.94		14.74				

Replicate 2

	Pl1	Ct mean	Rpl7	Ct mean	ΔCt	ΔΔCt	RQ ($2^{-\Delta\Delta Ct}$)
Scrambled	25.43	25.45	14.54	14.58	10.87	0.00	**1.00**
	25.54		14.57				
	25.38		14.62				
290-cluster inhibited	22.90	22.88	15.12	15.13	7.76	-3.12	**8.67**
	22.88		15.18				
	22.87		15.08				

Replicate 3

	Pl1	Ct mean	Rpl7	Ct mean	ΔCt	ΔΔCt	RQ ($2^{-\Delta\Delta Ct}$)
Scrambled	24.87	24.84	15.14	15.21	9.63	0.00	**1.00**
	24.85		15.22				
	24.79		15.26				
290-cluster inhibited	22.97	23.01	15.78	15.84	7.17	-2.46	**5.51**
	23.07		15.83				
	22.99		15.92				

E.

Source data Figure 4

Replicate 1

	Cdx2	Ct mean	Rpl7	Ct mean	ΔCt	ΔΔCt	RQ ($2^{-\Delta\Delta Ct}$)
Scrambled	21.95	21.87	15.62	15.63	6.24	0.00	**1.00**
	21.73		15.78				
	21.94		15.5				
322-cluster overexpressed	27.44	27.43	15.28	15.60	11.83	5.59	**0.02**
	27.3		15.96				
	27.56		15.56				

Replicate 2

	Cdx2	Ct mean	Rpl7	Ct mean	ΔCt	ΔΔCt	RQ ($2^{-\Delta\Delta Ct}$)
Scrambled	21.86	21.85	14.54	14.58	7.28	0.00	**1.00**
	21.88		14.57				
	21.82		14.62				
322-cluster overexpressed	27.63	27.60	15.12	15.13	12.47	5.20	**0.03**
	27.58		15.18				
	27.59		15.08				

Replicate 3

	Cdx2	Ct mean	Rpl7	Ct mean	ΔCt	ΔΔCt	RQ ($2^{-\Delta\Delta Ct}$)
Scrambled	21.15	21.20	16.29	16.27	4.93	0.00	**1.00**
	21.27		16.34				
	21.17		16.17				
322-cluster overexpressed	22.97	23.01	15.98	15.93	7.08	2.15	**0.23**
	23.07		15.95				
	22.99		15.87				

Replicate 1

	Eomes	Ct mean	Rpl7	Ct mean	ΔCt	ΔΔCt	RQ ($2^{-\Delta\Delta Ct}$)
Scrambled	20.68	20.79	16.6	16.68	4.11	0.00	**1.00**
	20.72		16.47				
	20.96		16.97				
322-cluster overexpressed	23.75	23.78	16.56	16.66	7.11	3.01	**0.12**
	23.71		16.6				
	23.87		16.83				

Replicate 2

	Eomes	Ct mean	Rpl7	Ct mean	ΔCt	ΔΔCt	RQ ($2^{-\Delta\Delta Ct}$)
Scrambled	21.22	21.19	15.29	15.39	5.79	0.00	**1.00**
	21.18		15.33				
	21.16		15.56				
322-cluster overexpressed	23.90	23.92	15.42	15.37	8.55	2.76	**0.15**
	23.97		15.50				
	23.88		15.18				

Replicate 3

	Eomes	Ct mean	Rpl7	Ct mean	ΔCt	ΔΔCt	RQ ($2^{-\Delta\Delta Ct}$)
Scrambled	21.17	21.25	15.78	15.68	5.58	0.00	**1.00**
	21.25		15.56				
	21.34		15.69				
322-cluster overexpressed	23.06	23.15	15.21	15.30	7.85	2.27	**0.21**
	23.15		15.34				
	23.25		15.36				

Replicate 1

	Esrrb	Ct mean	Rpl7	Ct mean	ΔCt	ΔΔCt	RQ ($2^{-\Delta\Delta Ct}$)
Scrambled	22.82	22.94	15.67	15.78	7.16	0.00	**1.00**
	22.97		15.7				
	23.02		15.97				
322-cluster overexpressed	27.94	27.87	15.6	15.68	12.19	5.04	**0.03**
	27.74		15.76				
	27.93		15.67				

Replicate 2

	Esrrb	Ct mean	Rpl7	Ct mean	ΔCt	ΔΔCt	RQ ($2^{-\Delta\Delta Ct}$)
Scrambled	21.76	21.70	15.91	15.92	5.78	0.00	**1.00**
	21.77		15.99				
	21.58		15.87				
322-cluster overexpressed	24.02	24.05	15.23	15.50	8.55	2.77	**0.15**
	24.12		15.18				
	24.00		16.08				

Replicate 3

	Esrrb	Ct mean	Rpl7	Ct mean	ΔCt	ΔΔCt	RQ ($2^{-\Delta\Delta Ct}$)
Scrambled	22.66	22.60	16.05	16.03	6.57	0.00	**1.00**
	22.54		16.04				
	22.59		15.99				
322-cluster overexpressed	26.87	26.91	15.79	15.68	11.23	4.66	**0.04**
	26.95		15.53				
	26.92		15.73				

Replicate 1

	Plf	Ct mean	Rpl7	Ct mean	ΔCt	ΔΔCt	RQ ($2^{-\Delta\Delta Ct}$)
Scrambled	23.58	23.71	14.92	14.97	8.73	0.00	**1.00**
	23.63		14.96				
	23.91		15.04				
322-cluster overexpressed	19.94	20.24	14.91	14.93	5.31	-3.42	**10.70**
	20.31		14.97				
	20.48		14.91				

Replicate 2

	Plf	Ct mean	Rpl7	Ct mean	ΔCt	ΔΔCt	RQ ($2^{-\Delta\Delta Ct}$)
Scrambled	22.86	22.82	15.63	15.56	7.26	0.00	**1.00**
	22.78		15.57				
	22.82		15.48				
322-cluster overexpressed	18.97	19.12	15.82	15.69	3.43	-3.83	**14.25**
	19.12		15.74				
	19.26		15.51				

Replicate 3

	Plf	Ct mean	Rpl7	Ct mean	ΔCt	ΔΔCt	RQ ($2^{-\Delta\Delta Ct}$)
Scrambled	23.7	23.62	15.34	15.40	8.22	0.00	**1.00**
	23.58		15.47				
	23.59		15.39				
322-cluster overexpressed	20.22	20.59	15.92	15.84	4.74	-3.48	**11.16**
	20.37		15.73				
	21.17		15.88				

Replicate 1

	Pl1	Ct mean	Rpl7	Ct mean	ΔCt	ΔΔCt	RQ ($2^{-\Delta\Delta Ct}$)
Scrambled	25.07	24.80	15.78	15.91	8.89	0.00	**1.00**
	24.6		15.97				
	24.72		15.97				
322-cluster overexpressed	21.12	21.19	15.94	15.90	5.29	-3.60	**12.10**
	21.3		15.79				
	21.16		15.97				

Replicate 2

	Pl1	Ct mean	Rpl7	Ct mean	ΔCt	ΔΔCt	RQ ($2^{-\Delta\Delta Ct}$)
Scrambled	24.42	24.58	15.52	15.58	9.00	0.00	**1.00**
	24.58		15.59				
	24.73		15.63				
322-cluster overexpressed	21.95	22.09	16.26	16.39	5.70	-3.30	**9.83**
	22.02		16.38				
	22.29		16.52				

Replicate 3

	Pl1	Ct mean	Rpl7	Ct mean	ΔCt	ΔΔCt	RQ ($2^{-\Delta\Delta Ct}$)
Scrambled	23.89	23.85	15.74	15.81	8.04	0.00	**1.00**
	23.71		15.83				
	23.96		15.87				
322-cluster overexpressed	19.89	19.81	16.01	15.94	3.87	-4.17	**18.04**
	19.75		15.99				
	19.78		15.82				

F.

For Figure 4F. CDX2, PLF and RPL7

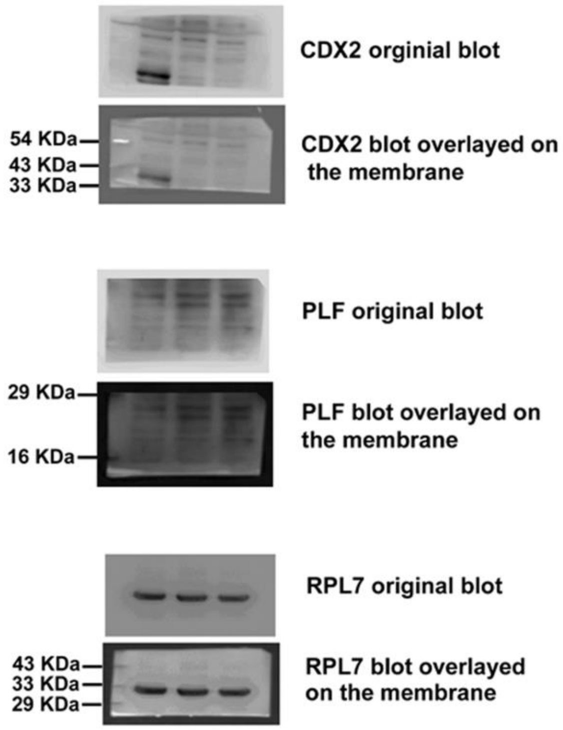

Supplement: Supplementary file 7 [file LSA-2020-00674_SdataF4.pdf]
