# Supplementary material for: MicroRNA regulation of murine trophoblast stem cell self-renewal and differentiation
Source: Life Sci Alliance. 2020 Sep 9;3(11):e202000674. doi: 10.26508/lsa.202000674 (PMC7494815; doi:10.26508/lsa.202000674)

Source data Figure 5

A.

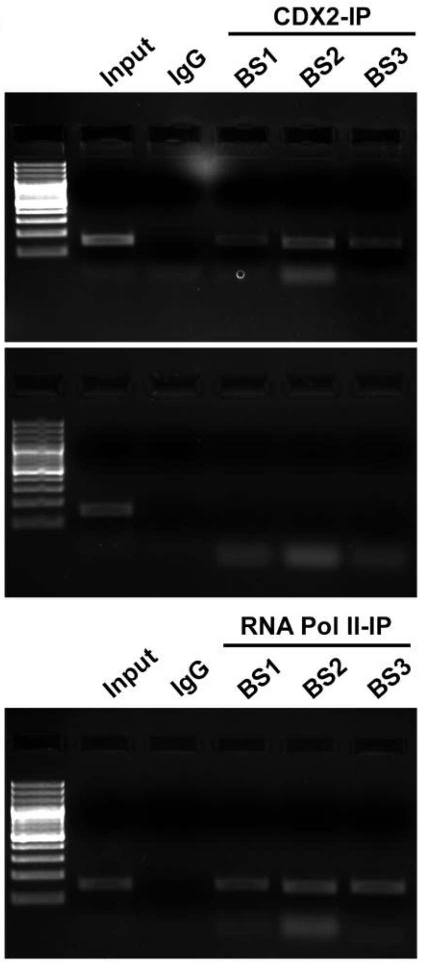

B.

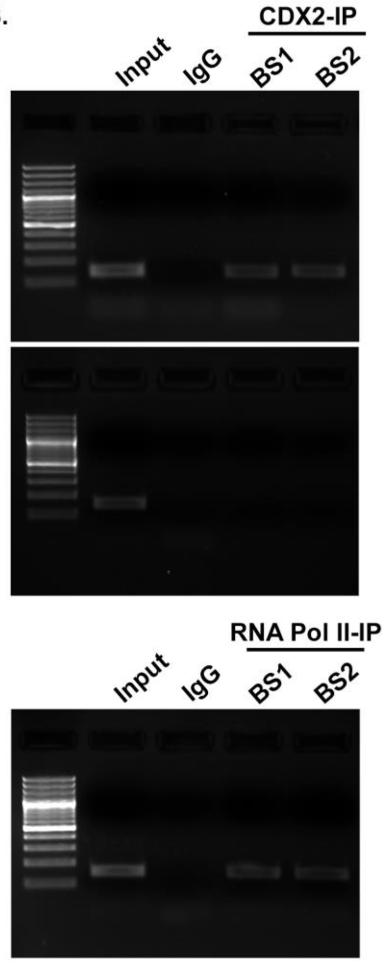

C.

**Replicate 1**							
	Cyclin D1	Ct mean	Rpl7	Ct mean	ΔCt	ΔΔCt	RQ ($2^{-ΔΔCt}$)
Scrambled	22.59	22.71	15.6	15.59	7.12	0.00	**1.00**
	22.64		15.52				
	22.91		15.66				
si-Cdx2	24.23	24.18	15.55	15.74	8.43	1.31	**0.40**
	24.1		15.7				
	24.2		15.98				
**Replicate 2**							
	Cyclin D1	Ct mean	Rpl7	Ct mean	ΔCt	ΔΔCt	RQ ($2^{-ΔΔCt}$)
Scrambled	21.7	21.39	15.42	15.45	5.94	0.00	**1.00**
	21.09		15.49				
	21.37		15.44				
si-Cdx2	22.83	22.55	15.86	15.80	6.75	0.81	**0.57**
	22.26		15.83				
	22.56		15.71				
**Replicate 3**							
	Cyclin D1	Ct mean	Rpl7	Ct mean	ΔCt	ΔΔCt	RQ ($2^{-ΔΔCt}$)
Scrambled	21	21.50	15.37	15.04	6.46	0.00	**1.00**
	21.21		14.92				
	22.28		14.83				
si-Cdx2	24.04	24.06	15.22	15.26	8.80	2.34	**0.20**
	23.87		15.18				
	24.28		15.39				

D.

For Figure 5D, CYCLIN D1 and RPL7

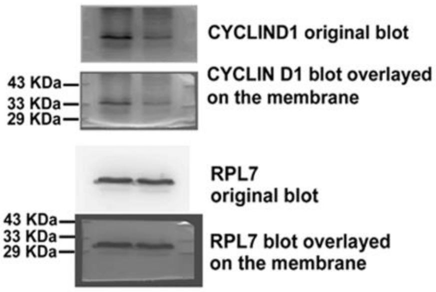

E.

Source data Figure 5

Replicate 1

	miR-291a-3p	Ct mean	U6snRNA	Ct mean	ΔCt	ΔΔCt	RQ ($2^{-\Delta\Delta Ct}$)
Scrambled	21.44	21.25	19.89	19.91	1.34	0.00	**1.00**
	21.37		19.86				
	20.94		19.97				
si-Cdx2	22.53	22.31	19.81	19.80	2.52	1.17	**0.44**
	22.14		19.74				
	22.27		19.84				

Replicate 2

	miR-291a-3p	Ct mean	U6snRNA	Ct mean	ΔCt	ΔΔCt	RQ ($2^{-\Delta\Delta Ct}$)
Scrambled	21.3	21.32	19.28	19.29	2.02	0.00	**1.00**
	21.28		19.32				
	21.37		19.28				
si-Cdx2	22.03	22.28	19.67	19.63	2.65	0.62	**0.65**
	22.35		19.59				
	22.46		19.64				

Replicate 3

	miR-291a-3p	Ct mean	U6snRNA	Ct mean	ΔCt	ΔΔCt	RQ ($2^{-\Delta\Delta Ct}$)
Scrambled	20.89	20.92	20.07	20.13	0.79	0.00	**1.00**
	20.92		20.17				
	20.96		20.15				
si-Cdx2	21.76	21.40	19.98	19.97	1.42	0.63	**0.65**
	21.18		20.04				
	21.25		19.9				

Replicate 1

	miR-291b-3p	Ct mean	U6snRNA	Ct mean	ΔCt	ΔΔCt	RQ ($2^{-\Delta\Delta Ct}$)
Scrambled	22.66	22.71	19.84	19.91	2.80	0.00	**1.00**
	22.66		19.9				
	22.81		19.98				
si-Cdx2	23.84	23.82	19.84	19.85	3.97	1.17	**0.44**
	23.69		19.85				
	23.94		19.86				

Replicate 2

	miR-291b-3p	Ct mean	U6snRNA	Ct mean	ΔCt	ΔΔCt	RQ ($2^{-\Delta\Delta Ct}$)
Scrambled	23.13	23.13	20.03	20.06	3.08	0.00	**1.00**
	23.09		20.1				
	23.18		20.04				
si-Cdx2	23.98	23.99	20.28	20.25	3.75	0.67	**0.63**
	24.03		20.19				
	23.97		20.27				

Replicate 3

	miR-291b-3p	Ct mean	U6snRNA	Ct mean	ΔCt	ΔΔCt	RQ ($2^{-\Delta\Delta Ct}$)
Scrambled	22.27	22.21	19.77	19.77	2.44	0.00	**1.00**
	22.11		19.83				
	22.24		19.71				
si-Cdx2	23.32	23.27	19.54	19.52	3.75	1.32	**0.40**
	23.26		19.49				
	23.24		19.53				

Replicate 1

	miR-292a-3p	Ct mean	U6snRNA	Ct mean	ΔCt	ΔΔCt	RQ ($2^{-\Delta\Delta Ct}$)
Scrambled	21.96	21.96	19.73	19.75	2.21	0.00	**1.00**
	21.95		19.75				
	21.98		19.77				
si-Cdx2	22.91	22.84	19.95	19.97	2.87	0.66	**0.63**
	22.67		19.97				
	22.94		19.98				

Replicate 2

	miR-292a-3p	Ct mean	U6snRNA	Ct mean	ΔCt	ΔΔCt	RQ ($2^{-\Delta\Delta Ct}$)
Scrambled	21.72	21.73	20.32	20.43	1.30	0.00	**1.00**
	21.78		20.47				
	21.68		20.49				
si-Cdx2	22.19	22.13	19.97	19.94	2.19	0.89	**0.54**
	22.12		19.93				
	22.08		19.91				

Replicate 3

	miR-292a-3p	Ct mean	U6snRNA	Ct mean	ΔCt	ΔΔCt	RQ ($2^{-\Delta\Delta Ct}$)
Scrambled	21.37	21.37	19.76	19.72	1.64	0.00	**1.00**
	21.32		19.69				
	21.41		19.72				
si-Cdx2	22.23	22.24	19.14	19.21	3.03	1.39	**0.38**
	22.28		19.2				
	22.21		19.28				

Replicate 1

	miR-294-3p	Ct mean	U6snRNA	Ct mean	ΔCt	ΔΔCt	RQ ($2^{-\Delta\Delta Ct}$)
Scrambled	20.94	20.88	19.32	19.20	1.68	0.00	**1.00**
	20.96		19.07				
	20.75		19.22				
si-Cdx2	21.77	21.67	18.63	18.59	3.08	1.40	**0.38**
	21.67		18.59				
	21.57		18.55				

Replicate 2

	miR-294-3p	Ct mean	U6snRNA	Ct mean	ΔCt	ΔΔCt	RQ ($2^{-\Delta\Delta Ct}$)
Scrambled	21.12	21.16	19.45	19.45	1.72	0.00	**1.00**
	21.28		19.46				
	21.09		19.43				
si-Cdx2	22.98	23.05	19.83	19.83	3.22	1.50	**0.35**
	23.06		19.79				
	23.11		19.87				

Replicate 3

	miR-294-3p	Ct mean	U6snRNA	Ct mean	ΔCt	ΔΔCt	RQ ($2^{-\Delta\Delta Ct}$)
Scrambled	21.75	21.66	19.34	19.39	2.28	0.00	**1.00**
	21.79		19.44				
	21.45		19.38				
si-Cdx2	22.95	23.01	19.25	19.21	3.80	1.52	**0.35**
	23.14		19.09				
	22.93		19.28				

Replicate 1

	miR-295-3p	Ct mean	U6snRNA	Ct mean	ΔCt	ΔΔCt	RQ ($2^{-\Delta\Delta Ct}$)
Scrambled	21.99	21.90	18.8	18.89	3.01	0.00	**1.00**
	21.91		18.74				
	21.79		19.13				
si-Cdx2	22.96	22.82	18.12	18.54	4.28	1.27	**0.41**
	22.75		18.7				
	22.75		18.81				

Replicate 2

	miR-295-3p	Ct mean	U6snRNA	Ct mean	ΔCt	ΔΔCt	RQ ($2^{-\Delta\Delta Ct}$)
Scrambled	22.22	22.26	19.32	19.29	2.98	0.00	**1.00**
	22.31		19.28				
	22.26		19.26				
si-Cdx2	24.88	24.84	20.17	20.11	4.73	1.76	**0.30**
	24.89		20.03				
	24.75		20.12				

Replicate 3

	miR-295-3p	Ct mean	U6snRNA	Ct mean	ΔCt	ΔΔCt	RQ ($2^{-\Delta\Delta Ct}$)
Scrambled	22.46	22.59	20.37	20.33	2.26	0.00	**1.00**
	22.58		20.35				
	22.73		20.28				
si-Cdx2	25.28	25.35	19.16	19.11	6.24	3.98	**0.06**
	25.4		19.03				
	25.36		19.14				

F.

Source data Figure 5

Replicate 1	miR-291a-3p	Ct mean	U6snRNA	Ct mean	ΔCt	ΔΔCt	RQ ($2^{-\Delta\Delta Ct}$)
FLAG	27.54	27.65	19.51	19.53	8.12	0.00	**1.00**
	27.62		19.54				
	27.8		19.55				
CDX2-OE	26.82	26.88	19.79	19.89	6.99	-1.13	**2.19**
	26.86		19.94				
	26.97		19.95				

Replicate 2	miR-291a-3p	Ct mean	U6snRNA	Ct mean	ΔCt	ΔΔCt	RQ ($2^{-\Delta\Delta Ct}$)
FLAG	27.24	27.32	18.86	18.80	8.52	0.00	**1.00**
	27.37		18.72				
	27.36		18.82				
CDX2-OE	26.09	26.09	18.76	18.84	7.25	-1.28	**2.42**
	26.12		19				
	26.05		18.76				

Replicate 3	miR-291a-3p	Ct mean	U6snRNA	Ct mean	ΔCt	ΔΔCt	RQ ($2^{-\Delta\Delta Ct}$)
FLAG	26.95	26.97	19.98	19.89	7.07	0.00	**1.00**
	26.93		19.87				
	27.02		19.83				
CDX2-OE	25.92	25.78	19.66	19.68	6.10	-0.97	**1.96**
	25.45		19.63				
	25.96		19.74				

Replicate 1	miR-291b-3p	Ct mean	U6snRNA	Ct mean	ΔCt	ΔΔCt	RQ ($2^{-\Delta\Delta Ct}$)
FLAG	26.74	26.86	19.49	19.52	7.34	0.00	**1.00**
	26.85		19.52				
	26.98		19.54				
CDX2-OE	26.34	26.45	19.87	19.92	6.53	-0.81	**1.75**
	26.46		19.95				
	26.54		19.93				

Replicate 2	miR-291b-3p	Ct mean	U6snRNA	Ct mean	ΔCt	ΔΔCt	RQ ($2^{-\Delta\Delta Ct}$)
FLAG	26.12	26.21	19.39	19.37	6.84	0.00	**1.00**
	26.11		19.34				
	26.41		19.39				
CDX2-OE	25.81	25.77	19.65	19.81	5.96	-0.88	**1.84**
	25.76		19.99				
	25.73		19.78				

Replicate 3	miR-291b-3p	Ct mean	U6snRNA	Ct mean	ΔCt	ΔΔCt	RQ ($2^{-\Delta\Delta Ct}$)
FLAG	26.52	26.51	19.61	19.63	6.89	0.00	**1.00**
	26.59		19.67				
	26.43		19.6				
CDX2-OE	25.92	25.68	19.63	19.41	6.27	-0.62	**1.54**
	25.72		19.33				
	25.39		19.27				

Replicate 1	miR-292a-3p	Ct mean	U6snRNA	Ct mean	ΔCt	ΔΔCt	RQ ($2^{-\Delta\Delta Ct}$)
FLAG	21.79	21.87	19.45	19.50	2.37	0.00	**1.00**
	21.88		19.55				
	21.95		19.5				
CDX2-OE	21.45	21.54	19.85	19.92	1.62	-0.75	**1.68**
	21.53		19.94				
	21.64		19.96				

Replicate 2	miR-292a-3p	Ct mean	U6snRNA	Ct mean	ΔCt	ΔΔCt	RQ ($2^{-\Delta\Delta Ct}$)
FLAG	21.23	21.28	18.89	18.86	2.42	0.00	**1.00**
	21.29		18.88				
	21.32		18.82				
CDX2-OE	20.83	20.82	19.03	18.96	1.86	-0.56	**1.47**
	20.78		18.87				
	20.85		18.98				

Replicate 3	miR-292a-3p	Ct mean	U6snRNA	Ct mean	ΔCt	ΔΔCt	RQ ($2^{-\Delta\Delta Ct}$)
FLAG	22.34	22.32	19.55	19.43	2.89	0.00	**1.00**
	22.27		19.37				
	22.35		19.38				
CDX2-OE	21.88	21.79	19.72	19.71	2.08	-0.81	**1.75**
	21.9		19.78				
	21.58		19.63				

Replicate 1	miR-294-3p	Ct mean	U6snRNA	Ct mean	ΔCt	ΔΔCt	RQ ($2^{-\Delta\Delta Ct}$)
FLAG	20.67	20.78	18.73	18.80	1.98	0.00	**1.00**
	20.78		18.74				
	20.9		18.94				
CDX2-OE	20.05	20.14	18.99	19.03	1.11	-0.87	**1.83**
	20.13		19.02				
	20.24		19.08				

Replicate 2	miR-294-3p	Ct mean	U6snRNA	Ct mean	ΔCt	ΔΔCt	RQ ($2^{-\Delta\Delta Ct}$)
FLAG	21.77	21.69	19.28	19.24	2.44	0.00	**1.00**
	21.56		19.28				
	21.73		19.17				
CDX2-OE	21.03	21.06	19.22	19.27	1.79	-0.65	**1.57**
	21.05		19.39				
	21.1		19.2				

Replicate 3	miR-294-3p	Ct mean	U6snRNA	Ct mean	ΔCt	ΔΔCt	RQ ($2^{-\Delta\Delta Ct}$)
FLAG	21.29	21.41	19.03	19.11	2.30	0.00	**1.00**
	21.38		19.17				
	21.57		19.13				
CDX2-OE	20.8	20.91	19.64	19.58	1.33	-0.97	**1.96**
	20.98		19.58				
	20.95		19.52				

Replicate 1	miR-295-3p	Ct mean	U6snRNA	Ct mean	ΔCt	ΔΔCt	RQ ($2^{-\Delta\Delta Ct}$)
FLAG	22.49	22.62	19.64	19.61	3.02	0.00	**1.00**
	22.61		19.58				
	22.77		19.6				
CDX2-OE	21.8	21.89	19.96	19.94	1.95	-1.07	**2.09**
	21.87		19.92				
	22.01		19.95				

Replicate 2	miR-295-3p	Ct mean	U6snRNA	Ct mean	ΔCt	ΔΔCt	RQ ($2^{-\Delta\Delta Ct}$)
FLAG	21.67	21.65	18.82	18.81	2.84	0.00	**1.00**
	21.55		18.73				
	21.74		18.89				
CDX2-OE	20.98	21.04	19.03	19.08	1.96	-0.88	**1.84**
	21.04		19				
	21.09		19.21				

Replicate 3	miR-295-3p	Ct mean	U6snRNA	Ct mean	ΔCt	ΔΔCt	RQ ($2^{-\Delta\Delta Ct}$)
FLAG	22.27	22.29	19.52	19.57	2.71	0.00	**1.00**
	22.21		19.57				
	22.38		19.63				
CDX2-OE	20.62	20.76	19.03	19.20	1.56	-1.15	**2.22**
	20.78		19.4				
	20.87		19.16				

Source data Figure 5

G.

Dual Luciferase Assay

Wild-type

		**Replicate 1**		**Replicate 2**		**Replicate 3**		**Replicate 4**	
		Mean OD	Rel.Luc.activity	Mean OD	Rel.Luc.activity	Mean OD	Rel.Luc.activity	Mean OD	Rel.Luc.activity
Scrambled	Firefly	7395848		9800932		10502088		10146996	
	Renilla	888258		917056		699471		596727	
	Normalized	8.32623855	1.000	10.687387	1.000	15.0143294	1.000	17.004419	1.000
miR-322-5p mimic	Firefly	5033299		5130506		4475373		3689315	
	Renilla	1133363		1401701		1399100		1301466	
	Normalized	4.44102993	0.533	3.6602	0.342	3.19875134	0.213	2.8347379	0.167
miR-503-5p mimic	Firefly	6915039		8971951		7605606		6814701	
	Renilla	943075		1427190		1252326		995071	
	Normalized	7.33243804	0.881	6.2864447	0.588	6.07318382	0.404	6.848457	0.403
miR-542-5p mimic	Firefly	7480058		10030614		9131519		6827893	
	Renilla	1082097		1412781		1363781		948208	
	Normalized	6.91255775	0.830	7.0999072	0.664	6.69573707	0.446	7.2008388	0.423

Mutated

		**Replicate 1**		**Replicate 2**		**Replicate 3**		**Replicate 4**	
		Mean OD	Rel.Luc.activity	Mean OD	Rel.Luc.activity	Mean OD	Rel.Luc.activity	Mean OD	Rel.Luc.activity
Scrambled	Firefly	4776771		7627275		7689735		6405466	
	Renilla	770805		1119180		991643		834751	
	Normalized	6.19711989	1.000	6.8150566	1.000	7.75453969	1.000	7.673505	1.000
miR-322-5p mimic	Firefly	5878430		4574449		4253986		4996001	
	Renilla	845130		746848		669455		678237	
	Normalized	6.95565179	1.122	6.1250067	0.899	6.35440171	0.819	7.3661581	0.960
miR-503-5p mimic	Firefly	6442415		6644299		7215024		4281428	
	Renilla	1148986		1059504		1151606		694811	
	Normalized	5.60704395	0.905	6.271141	0.920	6.26518445	0.808	6.1620038	0.803
miR-542-5p mimic	Firefly	6113826		3531484		6974800		6781499	
	Renilla	883756		579851		966786		1001376	
	Normalized	6.91800225	1.116	6.0903301	0.894	7.21441974	0.930	6.7721805	0.883

H.

Source data Figure 5

Replicate 1

	Cdx2	Ct mean	Rpl7	Ct mean	ΔCt	ΔΔCt	**RQ ($2^{-\Delta\Delta Ct}$)**
Scrambled	21.86	21.88	14.72	14.83	7.05	0.00	**1.00**
	21.92		14.9				
	21.86		14.87				
miR-322 mimic	24.69	24.53	14.55	14.57	9.95	2.90	**0.13**
	24.69		14.48				
	24.2		14.69				
miR-322 inhibitor	21.63	21.69	14.92	14.80	6.89	-0.16	**1.12**
	21.8		14.88				
	21.64		14.61				
miR-503 mimic	24.72	24.83	14.94	14.93	9.90	2.85	**0.14**
	24.7		14.94				
	25.07		14.9				
miR-503 inhibitor	21.46	21.34	14.76	14.89	6.45	-0.60	**1.51**
	21.36		14.96				
	21.2		14.94				
miR-542 mimic	22.34	22.38	14.75	14.79	7.58	0.53	**0.69**
	22.39		14.79				
	22.4		14.84				
miR-542 inhibitor	21.41	21.60	14.63	14.72	6.88	-0.17	**1.13**
	21.42		14.85				
	21.96		14.68				

Replicate 3

	Cdx2	Ct mean	Rpl7	Ct mean	ΔCt	ΔΔCt	**RQ ($2^{-\Delta\Delta Ct}$)**
Scrambled	22.83	22.74	15.98	15.98	6.77	0.00	**1.00**
	22.62		15.91				
	22.78		16.04				
miR-322 mimic	26.02	26.21	16.12	16.02	10.20	3.43	**0.09**
	26.37		16.01				
	26.25		15.92				
miR-322 inhibitor	22.47	22.44	15.97	15.97	6.47	-0.30	**1.23**
	22.3		15.99				
	22.54		15.95				
miR-503 mimic	25.02	24.91	15.74	15.71	9.20	2.43	**0.19**
	24.87		15.72				
	24.84		15.68				
miR-503 inhibitor	21.26	21.61	15.56	15.69	5.92	-0.85	**1.80**
	21.22		15.79				
	22.35		15.72				
miR-542 mimic	24.82	23.98	16.04	16.04	7.94	1.17	**0.44**
	23.22		16.2				
	23.9		15.89				
miR-542 inhibitor	22.9	22.78	15.29	15.20	7.58	0.81	**0.57**
	22.58		15.31				
	22.86		15				

Replicate 2

	Cdx2	Ct mean	Rpl7	Ct mean	ΔCt	ΔΔCt	**RQ ($2^{-\Delta\Delta Ct}$)**
Scrambled	22.14	22.27	15.34	15.38	6.89	0.00	**1.00**
	22.34		15.42				
	22.32		15.38				
miR-322 mimic	24.77	24.78	15.7	15.69	9.09	2.21	**0.22**
	24.84		15.76				
	24.73		15.6				
miR-322 inhibitor	21.6	21.80	15.54	15.48	6.32	-0.56	**1.48**
	21.92		15.54				
	21.89		15.36				
miR-503 mimic	24.98	25.01	15.33	15.42	9.59	2.70	**0.15**
	25.2		15.47				
	24.84		15.46				
miR-503 inhibitor	21.62	21.82	15.37	15.43	6.39	-0.50	**1.41**
	21.91		15.53				
	21.93		15.39				
miR-542 mimic	23.95	23.66	15.43	15.16	8.50	1.61	**0.33**
	23.9		15.48				
	23.12		14.56				
miR-542 inhibitor	22.32	22.51	15.54	15.54	6.97	0.08	**0.94**
	22.63		15.55				
	22.57		15.52				

I.

For Figure 5I, CDX2 and RPL7

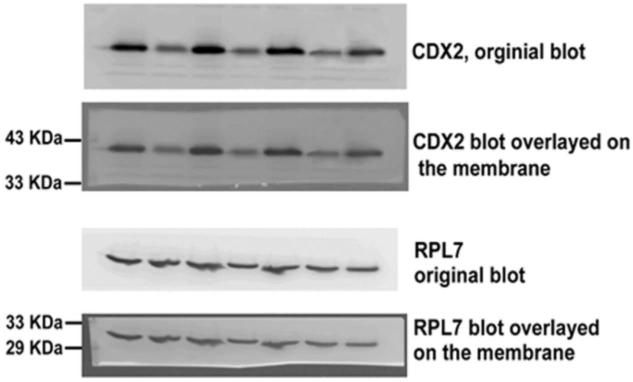

Supplement: Supplementary file 8 [file LSA-2020-00674_SdataF5.pdf]
